# PD-L1, TMB, MSI, and Other Predictors of Response to Immune Checkpoint Inhibitors in Biliary Tract Cancer

**DOI:** 10.3390/cancers13030558

**Published:** 2021-02-01

**Authors:** Alessandro Rizzo, Angela Dalia Ricci, Giovanni Brandi

**Affiliations:** Division of Oncology, IRCCS Azienda Ospedaliero-Universitaria di Bologna, 40138 Bologna, Italy; angeladalia.ricci@studio.unibo.it (A.D.R.); giovanni.brandi@unibo.it (G.B.)

**Keywords:** predictive biomarkers, PD-L1, TMB, immunotherapy, immune checkpoint inhibitors, biliary tract cancer, cholangiocarcinoma

## Abstract

**Simple Summary:**

Over the last decade, immune checkpoint inhibitors (ICIs) targeting programmed death 1 (PD-1), programmed death-ligand 1 (PD-L1), and cytotoxic T-lymphocyte antigen 4 (CTLA-4) have dramatically changed the therapeutic algorithm of several hematological and solid tumors. Of note, these agents have been also investigated in biliary tract cancer (BTC), reporting controversial results so far; in this setting, the role of ICIs is still to be established, and available data on immunotherapy in BTC patients are mainly limited to sub-analyses of basket trials and small single-arm studies. A crucial challenge is represented by the lack of validated predictive biomarkers, that could help identify responders to immunotherapy, a high unmet need in these immunologically “cold” malignancies where ICIs are still looking for their niche.

**Abstract:**

Biliary tract cancer (BTC) represents the second most frequently diagnosed primary liver cancer worldwide following hepatocellular carcinoma, and the overall survival of patients with unresectable disease remains poor. In recent years, the advent of immune checkpoint inhibitors (ICIs) has revolutionized the therapeutic landscape of several malignancies with these agents, which have also been explored in advanced BTC, as monotherapy or in combination with other anticancer agents. However, clinical trials evaluating ICIs in BTC have shown conflicting results, and the clinical benefit provided by immunotherapy seems limited to a small subgroup of BTC patients. Thus, the identification of reliable predictors of the response to immunotherapy represents a significant challenge in this setting. This review provides an overview of the available evidence on the biomarkers predictive of the response to ICIs in patients with advanced BTC, especially focusing on programmed death-ligand 1 (PD-L1), tumor mutational burden (TMB), microsatellite instability (MSI), and other emerging biomarkers.

## 1. Introduction

Biliary tract cancers (BTCs) encompass a group of aggressive, rare, and heterogeneous tumors arising in the bile duct system, comprising gallbladder cancer (GBC), ampulla of Vater cancer (AVC), and cholangiocarcinoma (CCA) [1,2]. CCA is classically divided into extrahepatic cholangiocarcinoma (eCCA), originating outside the liver and further subclassified into distal (dCCA) and perihilar cholangiocarcinoma (pCCA), and intrahepatic cholangiocarcinoma (iCCA), occurring within the liver parenchyma [3,4]. Of note, this classification—based on the anatomical location of BTCs within the biliary tree—mirrors remarkable differences in terms of tumor biology, molecular features, epidemiology, prognosis, and therapeutic approaches [5].

BTC represents the second most frequent hepatobiliary tumor following hepatocellular carcinoma (HCC), accounting for approximately 3% of all gastrointestinal malignancies worldwide [6]. Although BTCs have been traditionally considered rare tumors, their overall incidence has seen a remarkable increase over recent decades in most Western countries [7]. Radical surgery remains the only potentially curative treatment option, but unfortunately, most patients with BTC are diagnosed with advanced disease; moreover, a non-negligible proportion of BTCs initially considered resectable are subsequently found to be unresectable during exploratory laparotomy [8,9]. Additionally, even following surgical resection with negative tumor margins, distant and locoregional recurrence rates are high. In BTC patients with metastatic disease, systemic treatments represent the only potential therapeutic option. More than ten years after the publication of the landmark ABC-02 phase III trial, the combination of gemcitabine plus cisplatin remains the current standard of care in treatment-naïve patients [10,11]. According to the results of this study, the gemcitabine–cisplatin combination showed superior median overall survival (OS) compared to gemcitabine monotherapy (11.7 months versus 8.2 months, respectively; hazard ratio (HR), 0.64; 95% confidence interval (CI), 0.52–0.80; *p* < 0.001), with the ABC-02 establishing gemcitabine–cisplatin as the reference doublet [10,11]. Nonetheless, the limited survival benefit provided by systemic treatments has highlighted the need for more effective medical therapies in this setting [12].

The last decade has registered important advances in the understanding of the tumor biology of BTCs, as witnessed by the parallel development of novel treatment options and genomic sequencing, which has paved the way toward the identification of several possible therapeutic targets [13,14,15]. In fact, molecularly targeted therapies have been tested in BTC patients harboring specific druggable alterations, especially in iCCAs where agents targeting isocitrate dehydrogenase (IDH) mutations and fibroblast growth factor receptor (FGFR) aberrations have entered into clinical practice [16,17,18,19,20,21,22]; in addition, following the results observed in several hematological and solid malignancies, immune checkpoint inhibitors (ICIs) have been explored and are currently being investigated in BTC (Table 1) [23,24,25]. However, most BTC patients receiving ICIs as a monotherapy or in combination with other anticancer agents do not achieve response, and the mechanisms behind the variations in the response to immunotherapy in this setting have been poorly studied [26]. Based on these premises, the identification of biomarkers able to predict responses to ICIs and the understanding of resistance mechanisms in non-responders represent high unmet needs.

Herein, we provide an overview on the current knowledge regarding the predictive biomarkers of the response to ICIs in advanced BTCs, especially focusing on the role of programmed death-ligand 1 (PD-L1) expression, tumor mutational burden (TMB), mismatch repair deficiency (dMMR), high microsatellite instability (MSI-H), and DNA damage repair (DDR) gene mutations in this setting.

We performed research using PubMed/Medline, Cochrane Library, and Scopus with the keywords “biliary tract cancer” OR “cholangiocarcinoma” OR “intrahepatic cholangiocarcinoma” OR “extrahepatic cholangiocarcinoma” OR “gallbladder cancer” AND “immunotherapy” OR “immune checkpoint inhibitors” AND “PD-L1” OR “tumor mutational burden” OR “TMB” OR “MSI” OR “DDR” OR “DNA damage repair” OR “tumor microenvironment.” We selected pivotal registration studies. We also selected the most relevant and pertinent studies considering the quality of the studies in terms of their applicability, how they were conducted, statistical analysis, number of patients enrolled, and outcomes. For ongoing clinical trials, we searched in the clinicaltrials.gov database for currently recruiting and active trials, not simply recruiting trials, using the following keywords: “biliary tract cancer” OR “cholangiocarcinoma” OR “intrahepatic cholangiocarcinoma” OR “extrahepatic cholangiocarcinoma” OR “gallbladder cancer” AND “immunotherapy” OR “immune checkpoint inhibitors.” We restricted our research to phase I, II, or III trials focused on the metastatic/advanced setting.

## 2. PD-L1 Expression

The expression of PD-L1 assessed by immunohistochemistry has been shown to correlate with response to ICIs in several tumor types, including non-small cell lung cancer (NSCLC), gastric cancer, and urothelial carcinoma [27,28,29]. However, few data are available in BTC patients treated with ICIs so far. According to previous reports, PD-L1 expression has been reported to range from approximately 45% to 65% of immune cells within the tumor microenvironment and from 10% to 70% of tumor specimens [30,31]; in addition, PD-L1 expression by both intra-tumoral inflammatory or neoplastic cells has been related to tumor aggressiveness and worse survival [32,33]. First, Gani and colleagues evaluated the association between clinical outcomes and PD-L1 expression in 54 iCCA tumor samples, with PD-L1 assessed within the tumor front (TF) and in tumor-associated macrophages (TAMs) [34]. Of note, iCCAs expressing PD-L1 in the TF had a 60% reduced survival compared to PD-L1-negative patients [34]. Similar results were mirrored in more recent studies on other BTC subtypes, including pCCA and dCCA [30,31,32,33].

Regarding the predictive value of PD-L1 in this setting, interesting data may be extracted by the subgroup analyses of clinical trials assessing ICIs in advanced BTCs [35]. Among these, the KEYNOTE-028 phase Ib trial (Table 2) exclusively enrolled PD-L1-positive patients, with at least a 1% modified proportion score or interface pattern, which were treated with 10 mg/kg of pembrolizumab every two weeks [36]. According to the results of this study, after a median follow-up of 7.5 months, the overall response rate (ORR) was 13.0% in 23 previously treated BTC patients, with a median progression-free survival (PFS) and an overall survival (OS) of 1.8 and 5.7 months, respectively [36]. Similarly, the KEYNOTE-158 phase II trial investigated the role of 200 mg of pembrolizumab every three weeks in pretreated BTC patients with advanced disease [36]. At a median follow-up of 5.7 months in the overall population, a disappointing ORR of 5.8% was detected, with median PFS and OS of 2.0 and 7.4 months, respectively [36]. In a subgroup analysis especially focused on PD-L1-positive (*n* = 61) and PD-L1-negative (*n* = 34) BTC patients, the ORR was 6.6% in the first group and 2.9% in PD-L1-nonexpressers [36].

Another PD-1 inhibitor, nivolumab, was evaluated as a second-line treatment in 54 BTC patients with advanced disease in a recently published phase II trial [37]. In this study conducted by Kim and colleagues, the median PFS and OS in the overall population were 3.7 and 14.2 months, respectively, with an ORR of 22% and disease control rate (DCR) of 59% (Table 2). In 18 PD-L1-positive patients (≥1% of tumor cells expressing PD-L1 as a cutoff), a statistically significantly superior median PFS was observed compared to PD-L1-negative BTCs (10.4 months versus 2.3 months; HR, 0.23; 95% CI, 0.10–0.51; *p* < 0.001) [37]. In addition, a clinically meaningful superior median OS was observed in PD-L1-positive patients, despite not reaching statistical significance (not reached versus 10.8 months) [37].

Overall, the role of PD-L1 expression in predicting the response to ICIs in BTC is still to be defined. In addition, several methodological issues must be taken into account when discussing this topic in BTC, as well as in other tumor types [38,39]. Among these, the use of different PD-L1 assays, the lack of guidelines, the differences in scoring systems, and the discrepancy between metastatic and primary lesions have been suggested to be implied in reporting discordant results.

**Table 2:** Reported outcomes of single-agent immune checkpoint inhibitors in advanced biliary tract cancer (BTC).

Phase	Setting, (Type of BTC)	ICI	Agents Description	Number of Patients	Outcomes
Ib [36]	Second- or later-line (iCCA, eCCA, GBC)	Pembrolizumab	Pembrolizumab: PD-1 inhibitor	24 (all patients had PD-L1 ≥1%)	mPFS 1.8 months
					mOS 5.7 months
					ORR 13%
					SD rate 17%
II [36]	Second- or later-line (iCCA, eCCA, GBC)	Pembrolizumab	Pembrolizumab: PD-1 inhibitor	104 (61 patients had PD-L1 ≥1%)	mPFS 2.0 months
					mOS 7.4 months
					ORR 5.8% (6.6% in PD-L1+; 2.9% in PD-L1-)
II [40]	Second- or later-line (iCCA, eCCA, GBC)	Nivolumab	Nivolumab: PD-1 inhibitor	30	mPFS 1.4 months
					mOS 5.2 months
					PR rate 3%
II [37]	Second- or later-line (iCCA, eCCA, GBC)	Nivolumab	Nivolumab: PD-1 inhibitor	54	mPFS 3.7 months
					mOS 14.2 months
					ORR 22%
					DCR 50%
II [41]	Second- or later-line (iCCA, eCCA, GBC)	Durvalumab	Durvalumab: PD-L1 inhibitor	42	mPFS 1.5 months
					mOS 8.1 months
					PR rate 4.8%
I [42]	Second- or later-line (iCCA, eCCA, GBC)	M7824	M7824: PD-L1 inhibitor	30	mOS 12.7 months
					ORR 20%

AVC, ampulla of Vater cancer; DCR, disease control rate; eCCA, extrahepatic cholangiocarcinoma; GBC, gallbladder cancer; iCCA, intrahepatic cholangiocarcinoma; m, median; ORR, overall response rate; OS, overall survival; PD-1, programmed death 1; PFS, progression-free survival; PR, partial response; SD, stable disease.

## 3. TMB

**Figure 1:**
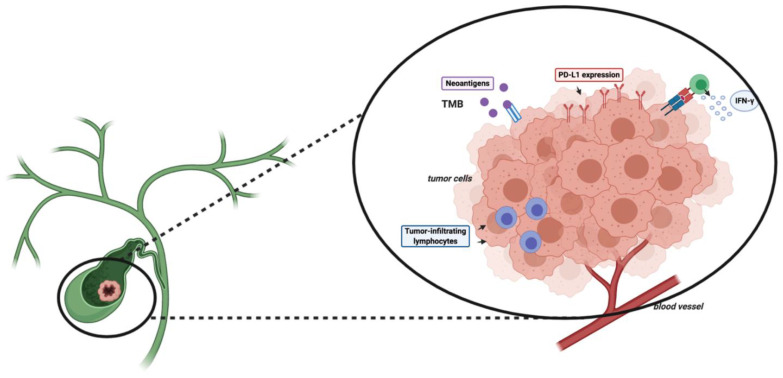
Schematic figure reporting some potential biomarkers of the response to immune checkpoint inhibitors (ICIs). Abbreviations: TMB, tumor mutational burden; PD-1, programmed death 1; PD-L1, programmed death-ligand 1.

Besides PD-L1 expression, TMB has been associated with responses to ICIs in several tumor types, despite this biomarker not having been prospectively validated yet [43]. TMB is commonly defined as the overall number of somatic nonsynonymous mutations per megabase (Mut/Mb), including frame-shift mutations, insertions, point mutations, and deletions [44,45]. The onset of these mutations is involved in the synthesis of abnormal proteins, which can act as neoantigens, activating antitumor responses (Figure 1) [46].

As in the case of PD-L1, TMB assessment is widely influenced by the kits and methods used that have been suggested to report different values in the same sample, and consequently, great attention and caution should be paid when comparing TMB values between studies using different methods [47,48]. In a genomic study by Weinberg and colleagues on 1502 BTCs, TMB was investigated in 352 tumor samples [49]. Based on a cutoff of 17 Mut/Mb, the authors observed that 4% of samples (14/352) had high TMB (TMB-H) [49]; of note, the proportion of TMB-H tumors was different in distinct BTC subgroups, with 5.8% (6/104), 3.5% (7/198), and 2% (1/50) of GBCs, iCCAs, and eCCAs, defined as TMB-H in this genomic report [49].

In terms of clinical responses to ICIs, data on TMB in BTC are sparse and anecdotal. A recently published study by Zhang and colleagues reported three BTC cases (one iCCA and two dCCAs) with TMB-H, which were treated with ICIs [50]; of note, all of these patients achieved response to immunotherapy, with two partial responses (PRs) and one case of complete response (CR) [50]. However, recent phase I and II clinical trials evaluating ICIs in advanced BTC did not report data in terms of TMB. Further studies are needed to understand the putative role of TMB in predicting the response to ICIs in BTC patients [50].

## 4. dMMR/MSI-H

In addition to PD-L1 and TMB, MSI is considered a potentially meaningful predictive biomarker of the response to ICIs, and has been associated with dMMR [51]. More specifically, MSI results in the accumulation of mutations, leading to the formation of neoantigens and the activation of antitumor immune responses [52,53].

Of note, the proportion of MSI-H status among BTC patients is controversial, as suggested by recent studies on this topic reporting conflicting results [54]. In fact, a landmark whole exome-sequencing report conducted by Nakamura and colleagues highlighted concurrent dMMR or MSI-H status in 36% of 260 BTC patients [55]. This proportion has been revised downward in a systematic review by Silva, estimating a proportion of dMMR and/or MSI-H of 10%, 5%–13%, and 5% in iCCA, eCCA, and GBC, respectively [56]. In addition, two reports by Weinberg and Winkelmann reported even lower proportions, with the former observing only 1% of dMMR by immunohistochemistry in 102 BTC specimens; conversely, the latter highlighted only seven cases of MSI-H/dMMR in a cohort of 352 BTCs (2%) [49,57].

As regards the predictive value of dMMR/MSI-H, few data are available so far. However, it is worth noting that in the previously discussed phase II trial on nivolumab monotherapy conducted by Kim and colleagues, all responders were microsatellite stable (MSS) [37]. Similarly, in the report by Zhang, the three BTC patients who achieved PR or CR with ICIs were all MSS [50]. In addition, the previously discussed KEYNOTE-158 and KEYNOTE-028 trials exploring the role of pembrolizumab in pretreated patients with advanced BTC reported an interesting finding: in fact, all responders to the ICI were microsatellite stable, adding further confusion on the putative role of MSI [36]. Of note, 95.2% (99/104) of the BTC patients enrolled in the KEYNOTE-158 were MSS, and pembrolizumab reported a disappointing response rate of 5.8%, as previously reported [36]. Conversely, only one MSI-H patient was included in the KEYNOTE-028 study, where no data on MSI were available in 37.5% of the enrolled BTCs [36].

Although the scarcity of data precludes from making a strong statement regarding the effective role of dMMR/MSI-H, available evidence seems to suggest an overall modest value of these biomarkers. Conversely, the evaluation of these biomarkers in concert with other potentially meaningful predictors could provide more useful information, as indicated by recently published studies on DDR, TMB, and MSI-H in this setting (Table 3).

## 5. DDR

Among the most promising predictive biomarkers of the response to immunotherapy, recent years have witnessed growing attention toward DDR gene alterations, based on preclinical and early phase clinical trials supporting this biological rationale [58,59]. Of note, DDR gene aberrations impair DNA damage repair processes, with subsequent accumulation of DNA damage [60]; in physiological conditions, genes such as poly (ADP-ribose) polymerase 1 and 2 (PARP1 and PARP2) play a key role in maintaining genomic stability and in avoiding the accumulation of these mutations, with the inhibition of these genes representing a timely topic in medical oncology [61].

Based on these premises, mutations in DDR genes have been recently studied in BTC, reporting interesting data on their possible role and their impact on modifying the responses to ICIs [62]. First, the proportion of DDR gene mutations in BTC has been reported to occur in approximately 30% of patients, while Breast Related Cancer Antigens (BRCA) mutations seem to fluctuate between 1% and 7%, according to previous reports [63,64,65]. A recently published study by Spizzo and colleagues analyzed tumor samples from 1292 BTC patients (iCCA, *n* = 746; eCCA, *n* = 189; GBC, *n* = 353) using next-generation sequencing [66]. Of note, BRCA mutations were observed in 3.6% of tumor samples, without showing significant differences according to tumor site [66]; in addition, an important finding of this report is the association between BRCA mutations, MSI/dMMR, and TMB-H, something that supports the evaluation of ICIs in a specific subgroup of BTC patients, with DDR gene mutations potentially representing biomarkers predictive of the response to immunotherapy [66,67].

Nonetheless, there is currently no consensus on the methods for testing DDR gene alterations and few data are available on the effective role of DDR gene mutations in BTC. Lastly, none of the recent studies investigating ICIs as a monotherapy or in combination with other anticancer agents in metastatic BTC have reported the number of patients harboring DDR aberrations; further studies are warranted in this direction to shed light on this promising—and still barely known—landscape.

## 6. TME

The tumor microenvironment (TME) represents another promising biomarker whose role as a predictor of the response to ICIs is under evaluation in several tumor types, with preclinical studies suggesting that TME could modify and modulate the host immune response against tumors [68,69,70]. As regards BTC, recent reports have highlighted that these hepatobiliary tumors are desmoplastic malignancies with the TME showing immunosuppressive innate immune cells, including tumor-associated macrophages and myeloid-derived suppressor cells [71,72,73]. In addition, the existence of distinct subgroups of tumors has been suggested, with immunologically “hot” and “cold” BTCs [74]. As regards the former, enhanced immune molecular expression, higher CD8+ T cell density, and a superior response rate to immunotherapy have been observed; in addition, this BTC subgroup seems to report increased PD-1 and PD-L1 expression, together with higher CD8+ T cell infiltration and enhanced granzyme B activity [75]. Conversely, the immune “cold” subgroup—which seems to represent the majority of these malignancies on the basis of the response rate observed in clinical trials assessing ICIs—presents a prevalence of immunosuppressive cells (e.g., tumor-associated macrophages and tolerogenic dendritic cells) and a non-T cell-infiltrated TME [75]. However, these results are still preliminary and offer an overall limited level of evidence.

## 7. Conclusions

ICIs are being assessed in advanced BTC, as a monotherapy or in combination with other anticancer agents, reporting controversial results so far; of note, most patients show disappointing clinical outcomes. Responses seem limited to a small percentage of BTCs. Unfortunately, the available data on the predictors of the response to ICIs in BTC are conflicting, and no single biomarker may select patients likely to benefit from this therapeutic approach. Moreover, we are aware that discussing potentially meaningful predictors could appear preliminary in a setting where immunotherapy is still trying to “find its way.” However, the identification of reliable predictors of the response to immunotherapy represents a compelling and urgent need in this aggressive malignancy with limited treatment options.

## Figures and Tables

**Table 1:** Ongoing phase I to III clinical trials evaluating immune checkpoint inhibitors in biliary tract cancer patients with advanced disease.

NCT Name	Phase	Setting (Type of BTC)	Arm A	Arm B	Agents Description	Primary Outcomes	Estimated Enrollment
NCT04066491	II/III	First-line (iCCA, eCCA, GBC)	Bintrafusp alfa (M7824) plus CisGem	Placebo plus CisGem	Bintrafusp alfa: first-in-class bifunctional fusion protein composed by a PD-L1 antibody fused with 2 extracellular domains of TGF-β receptor	DLTs OS	512
NCT03875235 (TOPAZ-1)	III	First-line (iCCA, eCCA, GBC)	Durvalumab plus CisGem	Placebo plus CisGem	Durvalumab: PD-L1 inhibitor	OS	757
NCT03260712	II	First-line (iCCA, eCCA, GBC)	Pembrolizumab plus CisGem		Pembrolizumab: PD-1 antibody	PFS at 6 months	50
NCT04300959	II	First-line (iCCA, eCCA, GBC)	Anlotinib plus sintilimab plus CisGem	CisGem	Anlotinib: TKI inhibiting PDGFR, FGFR, VEGFR and c-KIT kinase Sintilimab: PD-1 antibody	12-month OS rate	80
NCT03046862	II	First-line (AVC, iCCA, eCCA, GBC)	Durvalumab plus tremelimumab plus CisGem		Durvalumab: PD-L1 inhibitor Tremelimumab: anti-CTLA-4 agent	ORR	31
NCT03796429	II	First-line (iCCA, eCCA, GBC)	Toripalimab plus S-1 plus gemcitabine		Toripalimab: PD-1 antibody	PFS OS	40
NCT04172402	II	First-line (AVC, iCCA, eCCA, GBC)	Nivolumab plus S-1 plus gemcitabine		Nivolumab: PD-1 antibody	ORR	48
NCT04027764	II	First-line (iCCA, eCCA, GBC)	Toripalimab plus S-1 plus albumin paclitaxel		Toripalimab: PD-1 antibody	ORR	30
NCT03478488	III	First-line (iCCA, eCCA, GBC)	KN035 plus GEMOX	GEMOX	KN035: PD-L1 inhibitor	OS	390
NCT04191343	II	First-line (iCCA, eCCA, GBC)	Toripalimab plus GEMOX		Toripalimab: PD-1 antibody	ORR	20
NCT04003636 (KEYNOTE-966)	III	First-line (iCCA, eCCA, GBC)	Pembrolizumab plus CisGem	Placebo plus CisGem	Pembrolizumab: PD-1 antibody	PFS OS	788
NCT03937895	I/IIA	First- or later-line (iCCA, eCCA, GBC)	Pembrolizumab plus allogenic NK cell (SMT-NK)		Pembrolizumab: PD-1 antibody SMT-NK: allogenic natural killer cell	DLTs ORR	40
NCT03639935	II	Maintenance after platinum-based first-line chemotherapy (iCCA, eCCA, GBC)	Nivolumab plus rucaparib		Nivolumab: PD-1 antibody Rucaparib: PARP inhibitor	Proportion of patients alive and without radiological or clinical progression at 4 months	35
NCT03785873	Ib/II	Second-line (iCCA, eCCA, GBC)	Nivolumab plus 5-FU plus NalIri		Nivolumab: PD-1 antibody	DLTs PFS	40
NCT04298021	II	Second-line (AVC, iCCA, eCCA, GBC)	AZD6738 (ceralasertib) plus durvalumab	AZD6738 plus olaparib	AZD6738: ATR and ATM inhibitor Durvalumab: PD-L1 inhibitor Olaparib: PARP inhibitor	DCR	74
NCT03110328	II	Second-line (iCCA, eCCA)	Pembrolizumab		Pembrolizumab: PD-1 antibody	PFS OS Best overall response	33
NCT04211168	II	Second-line (iCCA, eCCA, GBC)	Toripalimab plus lenvatinib		Toripalimab: PD-1 antibody Lenvatinib: TKI	ORR AEs	44
NCT03797326	II	Second-line (AVC, iCCA, eCCA, GBC)	Pembrolizumab plus lenvatinib		Pembrolizumab: PD-1 antibody Lenvatinib: TKI	ORR AEs	600
NCT04010071	II	Second-line (AVC, iCCA, eCCA, GBC)	Toripalimab plus axitinib		Toripalimab: PD-1 antibody Axitinib: TKI	ORR PFS	60
NCT03704480 (IMMUNO-BIL)	II	Second-line (iCCA, eCCA, GBC)	Durvalumab plus tremelimumab	Durvalumab plus tremelimumab plus paclitaxel	Durvalumab: PD-L1 inhibitor Tremelimumab: anti-CTLA-4 agent	PFS	102
NCT03999658	II	Second- or later-line (AVC, iCCA, eCCA, GBC)	STI-3031		STI-3031: PD-L1 inhibitor	ORR	220
NCT03475953	I/II	Second- or later-line (AVC, iCCA, eCCA, GBC)	Avelumab plus regorafenib		Avelumab: PD-L1 inhibitor Regorafenib: TKI	RP2D	362
NCT03801083	II	Second- or later-line (AVC, iCCA, eCCA, GBC)	TILs		TILs: Tumor-Infiltrating Lymphocytes	ORR	59
NCT04057365	II	Second- or later-line (iCCA, eCCA, GBC)	Nivolumab plus DKN-01		Nivolumab: PD-1 antibody DKN-01: humanized monoclonal antibody against the DKK1 protein	ORR	30
NCT04298008	II	Third-line (AVC, iCCA, eCCA, GBC)	AZD6738 (ceralasertib) plus durvalumab		AZD6738: ATR and ATM inhibitor Durvalumab: PD-L1 inhibitor	DCR	26

This table includes ongoing clinical trials assessing immunotherapy as first-, second-, or later-line treatment. 5-FU: 5-fluorouracil; AEs, adverse events; ATM, ataxia-telangiectasia mutation; AVC, ampulla of Vater cancer; BTC, biliary tract cancer; CisGem, cisplatin plus gemcitabine combination; CTLA-4, cytotoxic T-lymphocyte antigen 4; DCR: disease control rate; DLTs, dose-limiting toxicities; eCCA: extrahepatic cholangiocarcinoma; FGFR, fibroblast growth factor receptor; GBC, gallbladder cancer; GEMOX, gemcitabine plus oxaliplatin; iCCA, intrahepatic cholangiocarcinoma; ORR, overall response rate; OS, overall survival; PARP, poly ADP ribose polymerase; PDGFR, platelet-derived growth factor receptor; PD-1, programmed death 1, PFS, progression-free survival; RP2D, recommended phase II dose; S-1: tegafur/gimeracil/oteracil; TILs: tumor infiltrating lymphocytes; TKI, tyrosine kinase inhibitor; VEGFR, vascular endothelial growth factor.

**Table 3:** Table reporting some possible advantages and disadvantages of the frequently used biomarkers for predicting the response to immunotherapy.

Pros/Cons	PD-L1	MSI-H/dMMR	TMB
**Advantages**	- Easy, low-cost, widely available	- Available in all solid malignancies - PCR or IHC for determining dMMR	- Available for the majority of tumor types - Simultaneous detection of other potential predictors
**Disadvantages**	- Multiple assays - Lack of standardization - Unknown optimum cutoff threshold - Positivity cutoff may vary depending on tumor type - Accuracy for predicting response appears variable	- Relatively rare finding - Best method to determine MSI status remains unclear	- Expensive and time-consuming - Unknown optimum cutoff threshold - Positivity cutoff may vary depending on tumor type - Accuracy for predicting response appears variable - The assessment requires high-quality DNA

dMMR, mismatch repair deficiency; IHC, immunohistochemistry; MSI-H, high microsatellite instability; PCR, polymerase chain reaction; PD-L1, programmed death-ligand 1; TMB, tumor mutational burden.

## Data Availability

Not applicable.
